# Upregulation of Metallothionein 1 G (MT1G) Negatively Regulates Ferroptosis in Clear Cell Renal Cell Carcinoma by Reducing Glutathione Consumption

**DOI:** 10.1155/2022/4000617

**Published:** 2022-09-27

**Authors:** Wu Zhang, Min Luo, Bingrui Xiong, Xiuheng Liu

**Affiliations:** ^1^Department of Urology, Renmin Hospital of Wuhan University, Wuhan 430060, Hubei, China; ^2^Department of Radiation and Medical Oncology, Zhongnan Hospital of Wuhan University, Wuhan 430061, China; ^3^Hubei Key Laboratory of Tumor Biological Behaviors, Wuhan 430061, China; ^4^Hubei Cancer Clinical Study Center, Wuhan 430061, China; ^5^Department of Anesthesiology, Zhongnan Hospital, Wuhan University, Wuhan, Hubei 430061, China

## Abstract

Ferroptosis is characterized by lipid peroxidation and iron accumulation, closely associated with clear cell renal cell carcinoma (ccRCC). It is of great significance for prognostic prediction and treatment of ccRCC to find biomarkers related to ferroptosis. We conducted several bioinformatic analyses using the transcriptome data and clinical information derived from online databases. Firstly, we identified the differentially expressed target genes in ccRCC. Then, *t* test and COX analysis were used to determine whether it was an independent prognostic factor combined with clinical information. String and gene set enrichment analysis (GSEA) were used to predict its function. Finally, we used ccRCC cells: 769-P and KAKI-1 in vitro to verify the regulation of target genes on cell proliferation apoptosis, iron metabolism, and GSH metabolism, which were used to judge the effect of target genes on ferroptosis. The study showed that MT1G is downregulated in ccRCC tissues compared with normal renal tissues. However, the ccRCC patients with higher expression relatively had higher malignancy and advanced stages. MT1G is an independent adverse factor for the prognosis of ccRCC. The protein interaction network analysis and GSEA showed that MT1G was closely related to GSH metabolism-related proteins (GSR) and lipid oxidation-related proteins (PLA2G2A). Samples with high expression of MT1G were enriched in “glutathione metabolism,” “oxidative phosphorylation,” and “proteasome,” whose function was involved in GSH metabolism and lipid peroxidation. The term associated with the occurrence and development of tumors included “P53 signaling pathway.” Furthermore, in vitro experiments showed that MT1G partially blocked ferroptosis induced by erastin and sorafenib-induced ccRCC cell lines (769-P and CAKI-1). The mechanism may be that MT1G affects ferroptosis by regulating GSH consumption in ccRCC cells. MT1G may be a negative regulator of ferroptosis in ccRCC cells and a biomarker of poor prognosis.

## 1. Introduction

Renal cancer is one of the most common tumors of the human urinary system, accounting for approximately 3% of all cancers and causing approximately 140,000 deaths worldwide each year[1, 2]. Clear cell renal cell carcinoma (ccRCC) is the most common pathological type, accounting for approximately 85% of all renal cancers, but has the lowest degree of malignancy [3]. Radical nephrectomy is generally used for the clinical treatment of ccRCC. However, the tumor is very likely to spread hematogenously, which occurs in almost 60% of patients [4, 5]. For these patients, targeted therapy and immunotherapy have become the first-line therapies [6]. Therefore, it is of great significance to further elucidate the pathophysiological mechanism of ccRCC and find new therapeutic drug targets to provide more effective and safe treatment strategies.

Ferroptosis is a recently defined form of regulatory cell death that was first proposed by Dixon in 2012 and is driven by iron-dependent lipid peroxidation [7]. Unlike autophagy and apoptosis, ferroptosis is iron- and reactive oxygen species (ROS)-dependent. The most notable feature of ferroptosis is the loss of plasma membrane selective permeability, which has been mainly attributed to substantial membrane lipid peroxidation and oxidative stress, which lead to certain cytological changes, including the reduction or disappearance of the mitochondrial crest, the rupture of the mitochondrial outer membrane, and the condensation of the mitochondrial membrane [8, 9]. Studies have shown that various physiological conditions and pathological stress responses can promote ferroptosis. Ferroptosis plays a key role in tumorigenesis by removing cells from an environment that lacks key nutrients or those that have been damaged by infection or environmental stress [10, 11]. Thus, ferroptosis is accepted as an adaptive feature to eliminate malignant tumor cells.

The kidney is an organ closely associated with iron metabolism and has a variety of biological functions, such as maintaining iron homeostasis and promoting hemoglobin synthesis through the formation of erythropoietin [12]. However, there are few studies on the mechanism of ferroptosis in ccRCC. Therefore, it is practical and meaningful to further study the complex mechanism of ferroptosis in ccRCC.

In this study, we used the public TCGA and ICGC databases to download transcriptome data and clinical data of patients with ccRCC. Then, from the FerrDb database (https://www.zhounan.org/ferrdb) (ferroptosis-related markers and regulatory factors diseases database) we obtained ferroptosis-related genes. Bioinformatics methods were used, and the ferroptosis gene metallothionein 1G (MT1G) was found to be strongly associated with the prognosis of ccRCC patients. MT1G encodes metallothionein and is responsible for metal ion homeostasis in cells. Its dysregulation has been reported to lead to various human tumors. Unfortunately, no studies have clarified the relationship between MT1G and ferroptosis in ccRCC. We confirmed the potential clinical value of MT1G by evaluating its differential expression, clinicopathological factors, and the prognosis of ccRCC patients. In addition, ccRCC cell lines were used to conduct in vitro experiments to explore the potential biological function of MT1G in ferroptosis in ccRCC patients.

## 2. Materials and Methods

### 2.1. Data Download and Analysis

In November 2020, we downloaded 259 ferroptosis-related genes obtained from FerrDb (https://www.zhounan.org/ferrdb) [13]. Moreover, we downloaded information on 539 ccRCC samples as well as relevant clinical data from The Cancer Genome Atlas (TCGA) database (https://www.cancer.gov/about-nci/organization/ccg/research/structural-genomics/tcga) [14]. Transcriptome data from the kidney tissues of 100 normal people were downloaded from the Genotype-Tissue Expression database (GTEx, https://www.gtexportal.org/home/index.html) [15]. We used Gene Expression Omnibus (GEO, https://www.ncbi.nlm.nih.gov/geo/) [16] to select two credible ccRCC-related datasets (GSE53757 and GSE66272) that have been cited in multiple studies as supplementary data.

### 2.2. Selection of MT1G Genes

The “limma” package of *R* version 4.2.0 was used to screen differentially expressed ferroptosis-related genes, with the following cutoff values: false discovery rate (FDR) < 0.05 and |log2 − fold change| > 1. Combined with the survival data, prognostic differentially expressed ferroptosis-related genes (PDEFRGs) were screened out by univariate Cox analysis and the Cox regression risk model. The final PDEFRGs were obtained by taking the intersection of the results filtered from the TCGA dataset and those filtered from the GEO dataset.

PDEFRGs were retrieved from the GEPIA (https://gepia.cancer-pku.cn/) [17], Human Protein Atlas (https://www.proteinatlas.org/) [18], and PubMed databases (https://pubmed.ncbi.nlm.nih.gov/). Genes with obvious differential expression that have not been studied in ccRCC were selected. Finally, MT1G was chosen as the target gene for subsequent studies. Furthermore, the expression of MT1G in ccRCC tissues and normal kidney tissues was analyzed.

### 2.3. Survival Analysis

Based on the median expression value, 539 ccRCC patients were allocated to the MT1G high expression group or the MT1G low expression group. The *R* software “survival” package, Kaplan–Meier method, and log-rank test were used to evaluate the effect of MT1G on the OS of ccRCC patients. In addition, the probability (*p*) values and 95% confidence intervals (CIs) were calculated, and a survival curve was plotted. Moreover, the package pROC was used to plot receiver operating characteristic (ROC) curves.

### 2.4. Relationship between the MT1G Expression Patterns and Clinicopathological Features

We selected clinicopathological data (age, sex, grade, TNM stage, infiltration depth (T), distant metastasis (M), and lymph node metastasis (N)) from the ccRCC tissue specimens from TCGA. After the exclusion of incomplete or defective clinical data, data from 246 patients were included for analysis. Independent sample *t*-tests and paired *t*-tests were used to identify correlations between MT1G expression levels and clinical-pathological parameters.

### 2.5. Identification of Independent Prognostic Factors

Univariate Cox regression analysis was performed to identify several prognostic factors, followed by multivariate Cox regression analysis to identify independent prognostic factors. All operations were performed by *R* version 4.2.0 software (“survival” and “survminer” packages).

### 2.6. Protein Interaction Network Analysis and Gene Set Enrichment Analysis (GSEA)

The STRING database (https://string-db.org/) [19] was used to explore the known and predicted correlations between protein interactions and MT1G expression patterns and to screen for proteins that interact with MT1G.

In Zhou's research [20], GSEA 4.1.0 software was used to identify MT1G-related signaling pathways.

### 2.7. Cell Culture

The ccRCC cell lines 769-P and CAKI-1 were purchased from Procell Life Science & Technology Co., Ltd., Wuhan, China, and were authenticated by STR profiling. There was no mycoplasma contamination. 769-P cells were cultured in RPMI-1640 (PM150110) containing 10% fetal bovine serum (FBS) (164210-500) and 1% penicillin *G* sodium/streptomycin sulfate. CAKI-1 cells were cultured in McCoy's 5A (PM150710) containing 10% FBS (164210-500) and 1% penicillin *G* sodium/streptomycin sulfate. The Petri dishes with these cells were placed at 37°C under 5% CO_2_ in a cell incubator.

### 2.8. Transfection

The MT1G overexpression vector and its negative control vector were constructed by Gene Pharma. After transfection with plasmids for 48 h, alterations in MT1G expression at the transcriptional and protein levels were evaluated by quantitative real-time PCR (qRT-PCR) and Western blotting analyses.

### 2.9. Drug Stimulation

The ccRCC cells were treated with 10 *µ*M erastin, sorafenib, and ferrostatin-1 for 24 hours. The untreated groups were given an appropriate amount of vehicle (0.1% DMSO).

### 2.10. CCK-8 Assay

Cell viability was examined by CCK-8 assay (MedChemExpress, China). Approximately 5000 cells were seeded in poly-l-lysine-coated96-well plates and subjected to various treatments as described above. CCK-8 solution (10 *μ*L/100 *μ*L) was added to each culture well, and neurons were incubated for 2 h at 37°C. Finally, the absorbance at 450 nm was measured with a microplate reader (cat. no. SpectraMax M2, Molecular Devices, Sunnyvale, CA, USA) at the same time each day [21].

### 2.11. Flow Cytometry (FCM) Analysis

769-P and CAKI-1 cells (1 × 10^6^ cells) were harvested, washed with PBS, and then centrifuged. Pellets were resuspended in 1 mL of DNA-staining solution, which contained 50 *μ*g/mL propidium iodide and 0.1 mg/mL RNase, and 10 *μ*L of permeabilization solution (Multisciences). The DNA content distribution was analyzed by flow cytometry (Beckman, cat. #FC500) after incubation in the dark at 37°C for 30 min. For cell apoptosis analysis, a FITC Annexin V Apoptosis Detection Kit I (BD Biosciences, USA) was used. BPH-1, WPMY-1, and RWPE-1 cells (1 × 10^6^ cells) were harvested and then stained with the FITC Annexin V Apoptosis Detection Kit I reagents according to the manufacturer's instructions [21].

### 2.12. Iron Assay

The intracellular iron concentration was measured with an Iron Assay kit (cat #BCA4355, Solarbio). Cells were lysed on ice, and then the supernatants were collected after centrifugation at 13,000*g* for 10 min. The supernatants were coincubated with 5 *μ*L of Iron Reducer solution at 37°C for 30 min. Subsequently, 100 *μ*L of Iron Probe was added for 1 hour of incubation at 37°C in the dark; thereafter, the absorbance was measured at 593 nm using a microplate reader.

### 2.13. Glutathione Assay

A total Glutathione Quantification Kit (Solarbio) was used to assess the relative GSH concentration in cell lysates. Cell samples (1.5 mL) were collected, 1 mL of lysate was added, the mixtures were ground thoroughly on ice, centrifuged at 8000 rpm for 10 min at 4°C, and the supernatant was collected. Then, 20 *μ*L of substrate working solution was added for 10 min of incubation at room temperature. The absorbance was then measured with a microplate reader at 412 nm.

### 2.14. Quantitative Real-Time PCR Analysis

According to the manufacturer's instructions, total RNA was isolated from frozen tissues and cells using TRIzol reagent (Invitrogen, Carlsbad, CA, USA) and quantitated at 260/280 nm using a NanoPhotometer spectrophotometer (IMPLEN, Westlake Village, CA, USA). Two micrograms of total RNA were reverse-transcribed to cDNA with the SuperScript II First-Strand Synthesis System according to the manufacturer's instructions (Invitrogen). qRT-PCR was performed to determine the level of mRNA expression of each gene of interest based on SYBR Green using a Bio-Rad CFX96 system (Hercules, CA, USA). The expression levels of the genes were normalized to the expression of GAPDH mRNA and compared by the 2^−ΔΔCT^ method. Values were normalized for amplified GAPDH alleles [21].

### 2.15. Western Blotting

Tissues and cells were lysed and ultrasonicated in RIPA reagent containing protease inhibitor and phosphatase inhibitor (Sigma–Aldrich) on ice for 30 min. The supernatant was collected after centrifugation at 14000 × *g* for 10 min at 4°C. Then, the protein concentration was measured by bicinchoninic acid assay. Protein extracts were isolated on sodium dodecyl sulfate–polyacrylamide (SDS–PAGE) gels and then transferred to polyvinylidene fluoride membranes (Millipore, Billerica, MA, USA) using a Bio-Rad wet transfer system. The membranes were then blocked in Tris-buffered saline with 0.05% Tween 20 (TBST) buffer containing 5% skim milk and incubated sequentially with primary and secondary antibodies. An enhanced chemiluminescence kit (Thermo Scientific Fisher, Waltham, MA, USA) was used to detect the bands.

### 2.16. Statistical Analysis

Statistical analyses were conducted using IBM SPSS Statistics for Windows version 20.0 (IBM Corporation, Armonk, NY, USA) and *R* version 4.2.0. All analyses were performed at least three times and represented data from three individual experiments. The data are expressed as the means ± standard deviations (SDs). A *p* value <0.05 was considered statistically significant. FDR < 0.05 and *p* < 0.01 were considered indicative of significant enrichment.

## 3. Results

**Figure 1:**
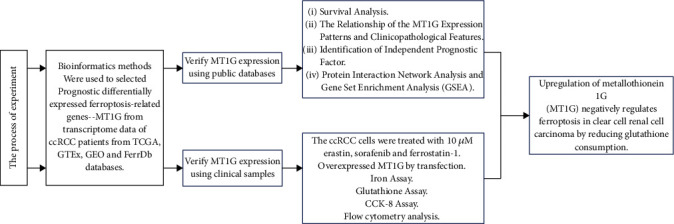
The flow chart of the experiment.

The study process is shown in Figure 1

### 3.1. Identification of PDEFRGs in ccRCC

**Figure 2:**
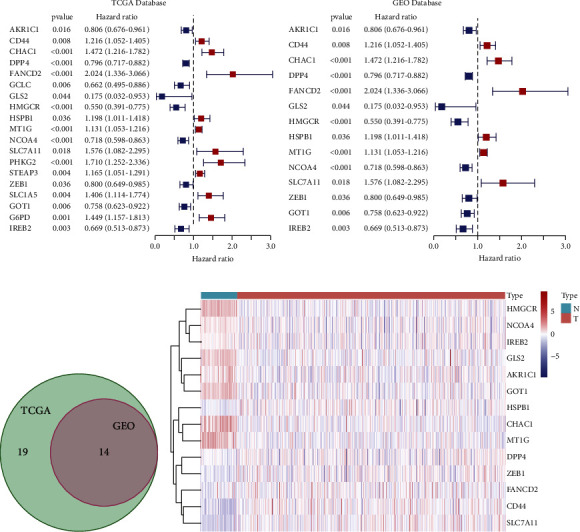
Prognostic differentially expressed ferroptosis-related genes of clear cell renal cell carcinoma (ccRCC) were selected from a public database. (a) Nineteen prognostic differentially expressed ferroptosis-related genes of ccRCC were selected from TCGA database. (b) Fourteen prognostic differentially expressed ferroptosis-related genes of ccRCC were selected from GEO database. (c) The intersection of two gene sets was taken. (d) The heatmap of 14 prognostic differentially expressed ferroptosis-related genes of ccRCC.

We analyzed the mRNA levels of 259 FRGs in 639 ccRCC samples (TCGA) and 100 normal renal tissue samples (GTEx). Nineteen PDEFRGs were screened out (10 upregulated and 9 downregulated) (Figure 2(a)). The same approach was applied to the GEO datasets, where 14 PDEFRGs were screened out (six upregulated and eight downregulated) (Figure 2(b)). Then, the data retrieved from the two databases were compiled (Figure 2(c)) [20]. In total, 14 PDEFRGs (AKR1C1, CD44, CHAC1, DPP4, FANCD2, GLS2, HMGCR, HSPB1, MT1G, NCOA4, SLC7A11, ZEB1, GOT1, and IREB2) were identified (Figure 2(d)) and were found to be significantly associated with the OS of ccRCC patients (all *p* < 0.05).

### 3.2. The Expression of MT1G in ccRCC

**Figure 3:**
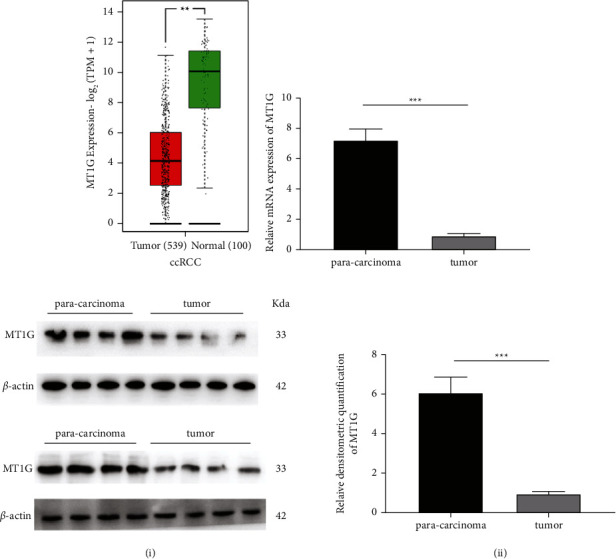
The expression of MT1G in clear cell renal cell carcinoma (ccRCC) and validation of tissue samples collected in clinic. (a) The expression level of MT1G in ccRCC was verified by using public databases. (b) The mRNA level of MT1G in ccRCC was verified by using clinical samples. (c) The protein level of MT1G in ccRCC was verified by using clinical samples (i). The relative densitometric quantification of MT1G (ii).

In this study, the transcriptome data of ccRCC patients from the TCGA database and GEO database and normal human kidney tissue from GTEx were used. By comparing MT1G expression levels in ccRCC tumor samples and normal kidney samples, it was found that MT1G was significantly downregulated in tumor tissues (*p* < 0.01) (Figure 3(a)). To verify the bioinformatics results, we collected eight ccRCC samples and their corresponding adjacent tissues for qRT-PCR analysis and Western blotting. The results showed that MT1G was significantly downregulated in tumor tissues at both the mRNA (Figure 3(b)) and protein levels (Figure 3(c)).

### 3.3. MT1G Is an Independent Poor Prognostic Factor of ccRCC and Is Associated with Clinicopathological Indices of ccRCC

**Figure 4:**
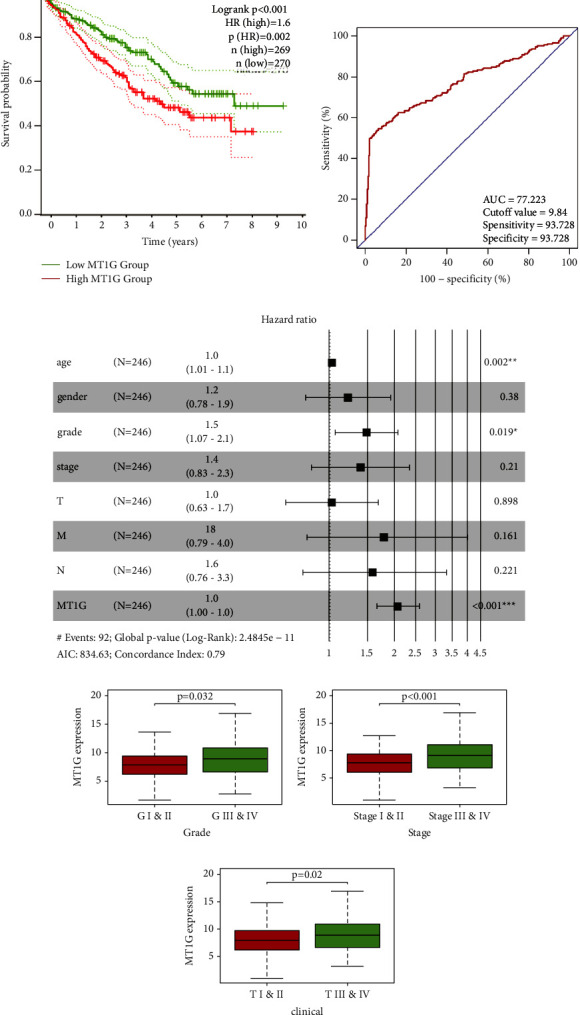
MT1G is an independent adverse prognostic factor in clear cell renal cell carcinoma (ccRCC) patients. (a) The survival curve of MT1G in ccRCC: the higher the expression level of MT1G, the shorter the survival time. (b) The receiver operating characteristic curve: AUC = 77.223. (c) Multivariate cox analysis showing the hazard ratios of different factors. The number of events for the number of tested factors was 92. The global *p* value (log-rank) was 2.4845e − 11, Akaike's information criterion was 834.63, and the concordance index was 0.79. (d) The expression level of MT1G was correlated with grade (*p*=0.032). (e) The expression level of MT1G was correlated with TNM stage (*p* < 0.001). (f) The expression level of MT1G was correlated with invasion depth (*p*=0.02).

A survival curve was plotted using the “survival” package of *R* version 4.2.0, the Kaplan–Meier method, and the log-rank test. The higher the expression of MT1G in ccRCC patients was, the shorter the survival time and the worse the prognosis of the patients (Figure 4(a)). The ROC curve showed that AUC = 77.223, sensitivity = 93.728, and specificity = 93.728 (Figure 4(b)). Furthermore, univariate and multivariate Cox regression analyses were performed to investigate whether the expression of MT1G could be an independent adverse prognostic factor in patients with ccRCC. As shown in Table 1, Cox univariate survival analysis indicated that age (*p*=0.01), grade (*p* < 0.001), TNM stage (*p* < 0.001), lymph node metastasis (*p* < 0.01), invasion depth (*p* < 0.001), distant metastasis (*p* < 0.001), and MT1G expression (*p* < 0.001) were important parameters affecting the duration of OS, while multivariate Cox survival analysis showed that age (*p* < 0.01), grade (*p* < 0.01), and MT1G expression (*p* < 0.001) were independent factors of poor ccRCC patient prognosis (Figure 4(c)).

A median gene expression value of 8.170 was used to stratify the 537 TCGA ccRCC patients into low and high expression groups. Analysis using TCGA clinical data and *R* version 4.2.0 showed that MT1G expression was correlated with grade (*p*=0.032) (Figure 4(d)), TNM stage (*p* < 0.001) (Figure 4(e)), and invasion depth (*p*=0.02) (Figure 4(f)).

### 3.4. Protein Interaction Network and GSEA of MT1G

**Figure 5:**
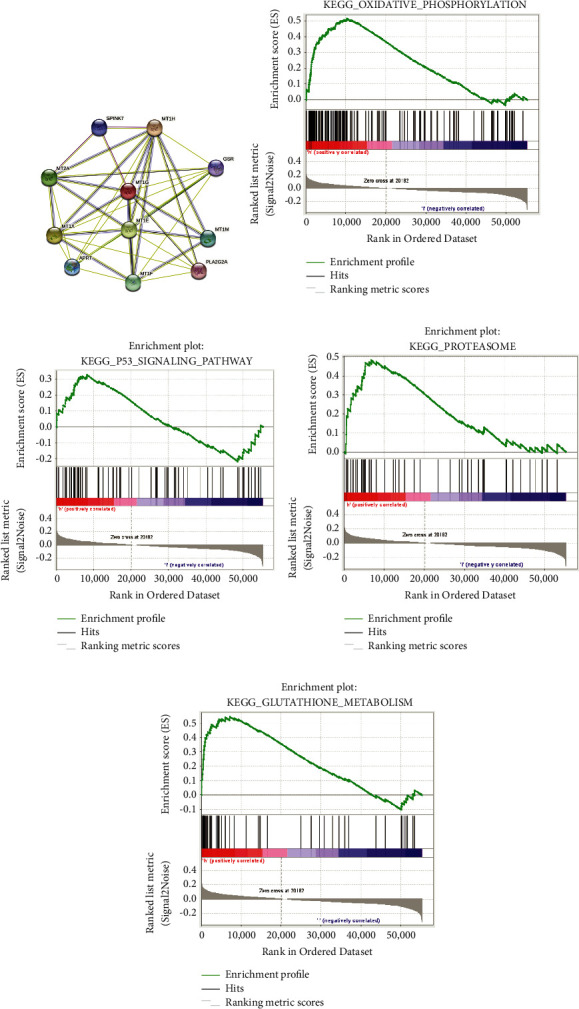
Protein interaction network and GSEA of MT1G. (a) An interaction network of the MT1G protein with other proteins (MT1H, MT1X, MT1E, MT2A, MT1F, MT1M, APRT, SPINK7, GSR, and PLA2G2A). The interaction network was obtained from the STRING database. (b) The MT1G upregulated samples were enriched in pathway of “Oxidative phosphorylation.” (c) The MT1G upregulated samples were enriched in pathway of “P53 signaling pathway.” (d) The MT1G upregulated samples were enriched in pathway of “Proteasome.” (e) The MT1G upregulated samples were enriched in pathway of “Glutathione metabolism.”

The STRING database was used to explore the known and predicted protein‒protein associations involving MT1G. The top 10 predicted functional partners were MT1H (0.913), MT1X (0.834), MT1E (0.785), MT2A (0.783), MT1F (0.783), MT1 M (0.554), APRT (0.539), SPINK7 (0.506), GSR (0.505), and PLA2G2A (0.501) (Figure 5(a)). GSEA identified 50 HCST-related signaling pathways that were upregulated in ccRCC, 13 of which were more notably enriched (NOM *p* < 0.05, FDR < 0.1, and NES > 1.5). As shown in Table 2, among these terms, “glutathione metabolism” (Figure 5(b)), “oxidative phosphorylation” (Figure 5(c)), and “proteasome” (Figure 5(d)), whose functions are involved in GSH metabolism and lipid peroxidation, were significantly enriched in the high MT1G expression group. Additionally, the “P53 signaling pathway” was included in the terms associated with the occurrence and development of tumors (Figure 5(e)).

### 3.5. MT1G Expression Increased ccRCC Cell Lines inhibiting Ferroptosis

**Figure 6:**
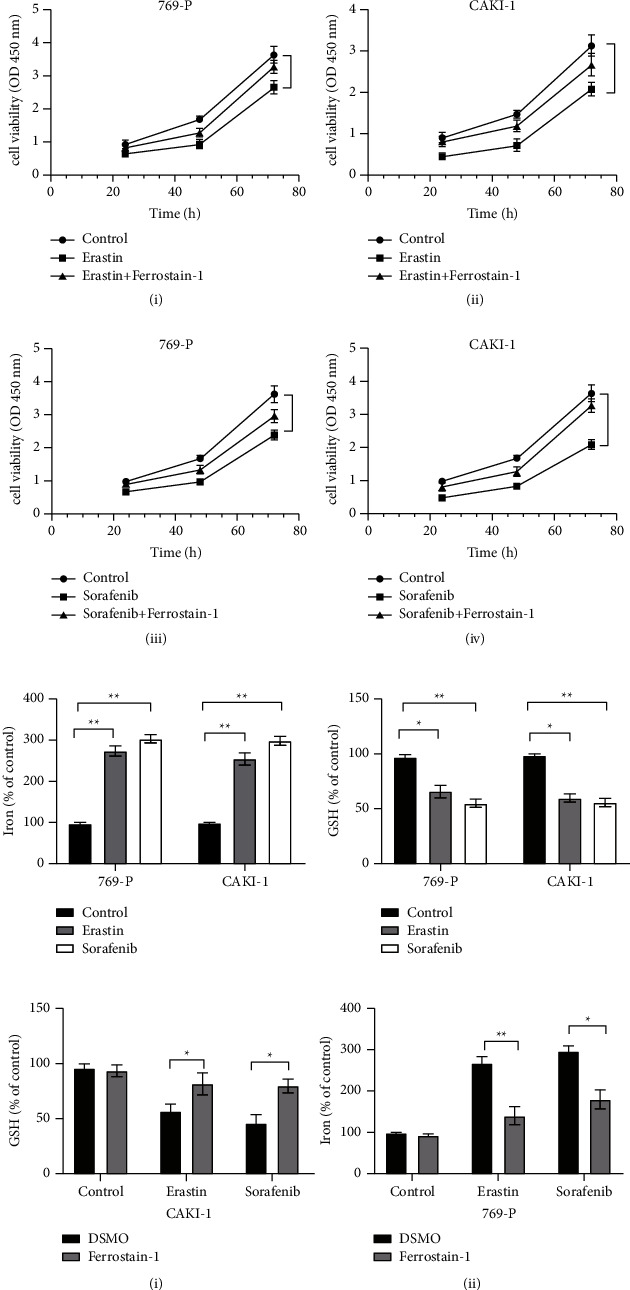
Erastin and sorafenib could induce ferroptosis in clear cell renal cell carcinoma cells. (a) Cell viability was determined by CCK-8 assay. Erastin could inhibit the growth of 769-P (i), while ferrostain-1 partially blocks this (iii). Sorafenib could inhibit the growth of CAKI-1 (ii), while ferrostain-1 partially blocks this (iv). (b) Iron concentration was measured with an Iron Assay kit. Erastin and sorafenib could increase the iron concentration of 769-P and CAKI-1. (c) GSH content in cell lysates was assayed by Total Glutathione Quantification Kit. Erastin and sorafenib could increase the GSH consumption of 769-P and CAKI-1. (d) The ferrostain-1 could recover the influence of erastin and sorafenib on the iron concentration and GSH consumption of CAKI-1 (i) and 769-P (ii).

In this study, after treatment with the classical ferroptosis inducers erastin and sorafenib, which are drivers of ferroptosis that block system xc−function, ferroptosis was induced in 769-P and CAKI-1 ccRCC cells. Erastin and sorafenib treatment led to the inhibition of cell viability (Figure 6(a)), the accumulation of reductive iron (Figure 6(b)), and depletion of GSH (Figure 6(c)). These changes were partially blocked by ferrostatin-1, a specific inhibitor of ferroptosis (Figure 6(d)).

**Figure 7:**
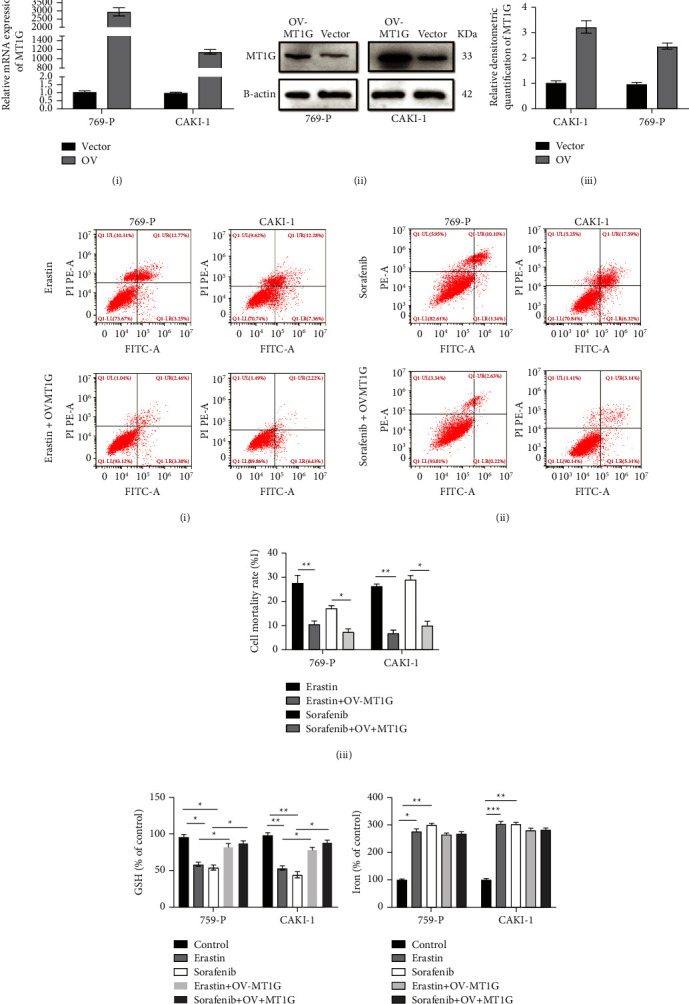
MT1G overexpression could inhibit ferroptosis of ccRCC. (a) QRT-PCR validated the efficiency of MT1G overexpression at transcriptional level in 769-P and CAKI-1 cells (i). Immunoblot assay (ii) and relative densitometric quantification of MT1G proteins (iii) in 769-P and CAKI-1 cells after overexpression of MT1G. (b) Flow cytometry analysis of the cell apoptosis in 769-P and CAKI-1 added erastin. 769-P and CAKI-1 added erastin after overexpression of MT1G for 48 hours (i). Flow cytometry analysis of the cell apoptosis in 769-P and CAKI-1 added sorafenib, and CAKI-1 added sorafenib after overexpression of MT1G for 48 hours (ii). Statistical analysis reveals the apoptotic rate (%) of 769-P and CAKI -1 after overexpression of MT1G (iii). (c) The overexpression of MT1G could recover the influence of erastin and sorafenib on the GSH consumption of 769-P and CAKI-1. (d) The overexpression of MT1G could not influence the iron concentration of 769-P and CAKI-1 added erastin and sorafenib.

To determine whether the increased expression of MT1G could affect ferroptosis in ccRCC cells, we successfully constructed MT1G overexpression vectors for transfection into both 769-P and CAKI-1 cells. The transfection efficiency was verified by qRT-PCR and Western blotting. The overexpression vector significantly elevated the expression level of MT1G (Figure 7(a)). As expected, the FCM results showed that the overexpression of MT1G partially eliminated the growth inhibition effects induced by erastin and sorafenib (Figure 7(b)). Moreover, GSH depletion in the two kinds of cells was also inhibited to some degree (Figure 7(c)). It is interesting to note that the level of redox-active iron in the cells did not change significantly (Figure 7(d)). Although the upregulation of MT1G seemed to have an effect similar to that of ferrostatin-1 by inhibiting ferroptosis in ccRCC cells induced by erastin and sorafenib, MT1G more likely regulated ferroptosis through GSH metabolism and lipid peroxidation rather than modulation of the level of redox-active iron.

## 4. Discussion

Clear cell renal cell carcinoma, the most common subtype of RCC, is an aggressive cancer characterized by abnormalities in GSH metabolism and lipid metabolism and is highly sensitive to GSH consumption [22, 23]. Many studies have shown that iron-related oxidative stress can lead to lipid oxidation and GSH consumption, resulting in different types of cell death, among which ferroptosis is typical [24]. MT1G is a metallothionein responsible for metal ion homeostasis in cells [25–27], and GSEA showed that it was associated with GSH metabolism in ccRCC. However, the effect of MT1G on ferroptosis in ccRCC and its potential prognostic or therapeutic value have not been investigated.

The transcriptome data of ccRCC and normal renal tissues, which were normalized and analyzed by the *R* version 4.2.0 “limma” package, were obtained from public databases (TCGA, GEO, and GTEx). Differential gene expression analysis showed that MT1G was significantly downregulated in ccRCC tissue samples compared with normal human kidney tissue samples. Alternatively, when further analyzing the correlation between MT1G expression and the clinicopathological factors in ccRCC patients, we found that MT1G was upregulated in tissues with a higher degree of malignancy and advanced tumor stage. Notably, MT1G could not be simply identified as a tumor suppressor gene or oncogenic gene in ccRCC, but MT1G seemed to play a dual role in the development and progression of tumors. After reviewing the literature, we found that MT1G plays important roles in metal homeostasis, the prevention of heavy metal toxicity and DNA damage, and oxidative stress [28, 29]. In recent years, some metalloenzymes have consequently received wide attention for tumor therapy due to its ROS generation ability and tumor cell killing ability [30]. Similarly, MT1G expression is thus essential for improving or eliminating the damage caused by heavy metals and free radicals to maintain redox homeostasis in cells. Therefore, upregulation of MT1G expression may be a strategy to prevent ccRCC. On the other hand, some studies have shown that high expression of MT1G is a prognostic factor for tumor progression and drug resistance in a variety of malignant tumors [31]. The expression of MT1G in hepatocellular carcinoma (HCC) was similar to that in ccRCC. MT1G upregulation could promote cancer progression by protecting HCC cells from sorafenib and inhibiting ferroptosis mediated by lipid peroxidation [32].

To further explore the potential role of MT1G in ccRCC, survival analysis was performed and combined with TCGA transcriptomic data and clinical information. Next, univariate and multivariate Cox regression analyses were used to determine whether MT1G was an independent prognostic factor. The results showed that ccRCC patients with a higher level of MT1G had a shorter survival time. MT1G could also be an independent adverse prognostic factor in ccRCC patients. Moreover, we collected tumor tissue and para-cancer tissue samples from eight patients with ccRCC who received surgical treatment. The qRT‒PCR and Western blot results showed that MT1G expression in tumor tissues was downregulated compared with that in the corresponding para-cancer tissues of patients. However, MT1G expression was upregulated in the tissues of patients in advanced stage or higher *T* stage compared with patients in a lower stage. The experimental results are consistent with those of our bioinformatics study.

Subsequently, we performed protein‒protein interaction analysis on MT1G using the STRING database. The results showed that the top 10 proteins associated with MT1G included MT1H, MT1X, MT1E, MT2A, MT1F, MT1 M, APRT, SPINK7, GSR, and PLA2G2A. In addition to the same family of metallothioneins, glutathione reductase (GSR) has been reported to be correlated with GSH metabolism and ferroptosis in tumor cells [33]. Regular metabolism in cells is capable of self-protection by converting the produced ROS into O_2_ or H_2_O via antioxidant mechanism. Such defense systems are mainly comprised of enzymes (GSH-Px, superoxide dismutase, and catalase), and reducing agents (cysteine, vitamin C, and GSH) [34]. According to previous studies, phospholipase A2 (PLA2G2A) might participate in cell membrane lipid oxidation [35]. The GSEA results explained that the pathways of “glutathione metabolism,” “oxidative phosphorylation,” and “proteasome” were enriched in MT1G-upregulated samples and were involved in GSH metabolism and lipid peroxidation. Moreover, the “P53 signaling pathway” was among the terms associated with the occurrence and development of tumors. These findings suggested that MT1G may regulate ferroptosis by affecting GSH metabolism and lipid peroxidation in ccRCC.

We next conducted in vitro experiments to verify the above bioinformatic results. Our results showed that erastin and sorafenib induced ferroptosis in 769-P and CAKI-1 cells in vitro. MT1G upregulation partially blocked the oxidative stress and ferroptosis induced by erastin and sorafenib, which was similar to treatment with ferrostatin-1. Therefore, we concluded that MT1G was involved in ferroptosis in ccRCC and acted as an inhibitor of ferroptosis. However, we found no significant differences in the levels of redox-active iron in MT1G-overexpressing 769-P and CAKI-1 cells after erastin and sorafenib treatment. Combined with our bioinformatics analysis, we found that MT1G was closely related to glutathione reductase. Branislav El.'s study also indicated that MT1G might affect GSH consumption by regulating GSH biosynthesis [36]. Therefore, we speculated that MT1G was likely to influence ferroptosis by regulating GSH consumption in ccRCC.

The inhibitory effect of MT1G on ferroptosis requires further study to confirm whether the same mechanism can also play a role in vivo and the specific mechanism by which MT1G regulates GSH metabolism.

Overall, our study identified that the ferroptosis-related gene MT1G was significantly downregulated in the tumor tissues of ccRCC patients. The expression of MT1G in normal human renal tissue may be essential for the elimination of heavy metals and free radicals to maintain cellular redox homeostasis. In ccRCC, the relative upregulation of MT1G may lead to tumor progression to the advanced stage and resistance to sorafenib, which might contribute to the effect of MT1G on ferroptosis by regulating GSH consumption. Therefore, MT1G, which is related to ferroptosis, has the potential to become a new prognostic biomarker and therapeutic target in of ccRCC.

## Figures and Tables

**Table 1:** Univariate analysis of the prognostic factors in ccRCC patients using a cox regression model.

Parameters OS	Univariate analysis		Multivariate analysis	
	HR (95% CI)	*p*	HR (95% CI)	*p*
MT1G expression high vs. low	2.251 (1.497∼3.003)	<0.001	1.889 (1.596∼2.173)	<0.001
Age ≥ 65 vs. <65	1.022 (1.005∼1.041)	0.012	1.032 (1.012∼1.053)	0.002
Female vs. male	1.013 (0.666∼1.541)	0.951	1.222 (0.781∼1.913)	0.380
TMN stage III/IV vs. I/II	1.862 (1.541∼2.251)	<0.001	1.394 (0.829∼2.342)	0.210
Grade G1/2 vs. G3/4	2.242 (1.682∼2.988)	<0.001	1.486 (1.066∼2.071)	0.019
Invasion depth T1/2 vs. T3/4	1.943 (1.538∼2.456)	<0.001	1.033 (0.633∼1.685)	0.898
Lymph node metastasis	2.932 (1.516∼5.668)	0.001	1.582 (0.759∼3.296)	0.221
Distant metastasis	4.073 (2.634∼6.300)	<0.001	1.786 (0.793∼4.020)	0.161

**Table 2:** GSEA pathways upregulated due to high expression of MT1G.

GS 〈br〉 follow link to MSigDB	ES	NES	*p*	FDR
KEGG_GLUTATHIONE_METABOLISM	0.56	2.05	0.001	0.008
KEGG_OXIDATIVE_PHOSPHORYLATION	0.51	1.84	<0.001	0.002
KEGG_PROTEASOME	0.48	1.76	<0.001	<0.001
KEGG_P53_SIGNALING_PATHWAY	0.39	1.70	<0.001	<0.001

## Data Availability

Publicly available datasets were analyzed in this study. The data can be found in the following link: https://portal.gdc.cancer.gov/?tdsourcetag=s_pcqq_aiomsg, https://www.ncbi.nlm.nih.gov/geo/, http://www.zhounan.org/ferrdb, https://www.gtexportal.org/home.

## Abbreviations

ccRCC: Clear cell renal cell carcinoma
TCGA: The Cancer Genome Atlas
GEO: Gene expression omnibus
GTEx: The genotype-tissue expression
FerrDb: The database of ferroptosis regulators and markers and ferroptosis-disease associations
FRG: Ferroptosis-related gene
PDEFRG: Prognostic differentially expressed ferroptosis-related genes
GSEA: Gene set enrichment analysis
OS: Overall survival.
